# Performance of point‐of‐care tests for diagnosing feline immunodeficiency virus infection in captive African lions ( *Panthera leo* )

**DOI:** 10.1111/avj.70137

**Published:** 2026-09-09

**Authors:** SE Jobbins, ME Westman, K Vinette Herrin, T Meili, R Hofmann‐Lehmann, JM Norris

**Affiliations:** ^1^ Sydney School of Veterinary Science, Faculty of Science The University of Sydney, Sydney, New South Wales 2006 Australia; ^2^ The Sydney Institute for Infectious Diseases, The University of Sydney, Sydney, New South Wales 2006 Australia; ^3^ Taronga Wildlife Hospital, Taronga Zoo Bradleys Head Rd, Mosman, New South Wales 2088 Australia; ^4^ Clinical Laboratory, Department of Clinical Diagnostics and Services, and Center for Clinical Studies, Vetsuisse Faculty The University of Zurich, Zurich CH‐8057 Switzerland

**Keywords:** African lion, diagnosis, lentivirus, retrovirus, serology, veterinary science, Western blot

## Abstract

Point‐of‐care (PoC) antibody (Ab) testing forms an integral component of feline immunodeficiency virus (FIV) diagnosis in both domestic and non‐domestic felids, with test kits designed for use in domestic cats frequently extrapolated for use in their wild counterparts. The SNAP® FIV/FeLV Combo lateral flow ELISA is commonly used to assign FIV infection status in wild and captive African lions (*Panthera leo*). The aim of this study was to investigate the performance of four other commercially available PoC FIV antibody test kits in African lions held in Australian zoos, when compared to SNAP® as a quasi‐reference test. To first validate the use of SNAP®, a subset of samples from SNAP® FIV‐positive (*n* = 3) and SNAP® FIV‐negative (*n* = 23) lions were evaluated with Western blotting, with perfect agreement demonstrated (Cohen's kappa coefficient = 1). Forty‐eight serum samples from SNAP® FIV‐positive (*n* = 16) and SNAP® FIV‐negative (*n* = 32) lions were then assessed with Anigen Rapid®, Witness®, VetScan® Rapid and SensPERT® FIV Ab tests. No seropositivity was observed in FIV‐infected lion sera using Anigen Rapid® or Witness® (Se 0%, Sp 100%), which utilise domestic cat FIV (FIV‐Fca) transmembrane glycoprotein gp40 as their capture antigen. In contrast, VetScan® Rapid, which employs FIV‐Fca capsid protein p24 as its capture antigen, demonstrated excellent performance (Se 100%, Sp 96.9%). The presence of p24 as a capture antigen did not guarantee the performance of an assay, as SensPERT®, which also features p24 as its capture antigen, was significantly less sensitive than VetScan® Rapid (Se 25%, Sp 100%; *p* < 0.001). Our findings have important implications for captive threatened felids, where timely, accurate diagnosis and careful management are critical for minimising retroviral transmission. We recommend using SNAP® or VetScan® Rapid FIV Ab tests for accurate diagnosis of FIV infection in African lions; however, the original VetScan® Rapid FIV test has been unavailable in Australia since 2019.

Feline immunodeficiency virus (FIV) is a lentivirus which was first discovered in domestic cats in 1986^1^ and is now identified globally in both wild and domestic felid populations.^2^ A member of Family *Retroviridae*, the single‐stranded RNA virus is reverse‐transcribed into cDNA and randomly inserted into the host genome, producing life‐long infection. FIV is thought to have originated in Africa since the late Pliocene epoch some 3.6–2.6 million years ago and has evolved to be highly host‐specific, with large genomic differences among its species‐specific strains.^3^ It is proposed that FIV infected an ancestral feline host, evolving with the diverging species' to produce the monophyletic, species‐specific clades seen today.^4^ At least 31 non‐domestic felid species are capable of becoming infected with FIV,^5^ with FIV subtype nomenclature reflecting this species‐specificity, for example, FIV‐Fca (domestic cat, *Felis catus*), FIV‐Ple (African lion, *Panthera leo*) and FIV‐Pco (puma, *Puma concolor*). FIV is endemic among non‐domestic *Felidae* in Africa and the Americas, but is rare in Asian species.^3^, ^6^ FIV is transmitted by close social contact – typically inoculation of bite wounds with virus‐laden saliva during fighting^7^; however, perinatal transmission between infected queens and their young has also been reported.^8^

Infection with FIV has been associated with serious illness in both domestic and wild cats, including progressive immune dysfunction and increased susceptibility to secondary infections, anaemia, stomatitis, renal insufficiency, neurological dysfunction and neoplasia^1^, ^9^, ^10^, ^11^, ^12^, ^13^, ^14^; however, not all investigators have identified increased morbidity and/or mortality in FIV‐infected individuals.^15^, ^16^, ^17^, ^18^ Indeed, management and housing conditions appear to play an important role in disease outcome in domestic cats,^18^ and in some non‐domestic felids, extensive host–virus co‐evolution is thought to have led to viral attenuation and/or host resistance to clinical illness.^19^ Nonetheless, it is likely that the multifactorial nature of disease, including host signalment, husbandry conditions, ecological conditions, variation in pathogenicity of locally circulating FIV serovars, as well as the prevalence and potential for exposure to opportunistic pathogens all interplay to produce the variable outcomes observed among both domestic and wild FIV‐infected felids. Importantly, pathological manifestations of immunodeficiency and chronic disease have been observed in captive^11^, ^13^ and wild^12^ FIV‐infected lions, highlighting the importance of accurate diagnosis for management of this threatened species.

For many years, the mainstay for FIV diagnosis in domestic cats has been point‐of‐care (PoC) antibody (Ab) tests, because they are accurate, inexpensive, easy to use and produce results quickly.^20^ Independent testing, however, has demonstrated the importance of test validation, with results from different brands of FIV kits manufactured by different companies not always comparable.^20^, ^21^, ^22^, ^23^ Alternative diagnostic modalities including CD4:CD8 T‐lymphocyte ratios, in situ hybridisation, Western blotting (WB) and virus isolation are available^10^, ^24^; however, these are more commonly used in research than in clinical practice.

A plethora of laboratory‐based assays have been utilised to assess FIV infection status in African lions, including WB,^11^, ^25^, ^26^, ^27^, ^28^ ELISA,^28^, ^29^, ^30^, ^31^, ^32^ nucleic acid amplification (PCR),^11^, ^13^, ^25^, ^31^, ^32^, ^33^, ^34^ flow cytometry^34^ and immunofluorescence.^26^, ^27^ Given the extensive sequence variation and highly monophyletic nature of FIV strains, the estimated disease prevalence for a population can vary significantly depending on the assay and, if applicable, antigen used.^27^, ^35^ The SNAP® FIV/FeLV Combo, which uses FIV‐Fca capture antigens p15 (matrix protein) and p24 (capsid protein), has been widely utilised to detect FIV infection in a range of non‐domestic felids,^12^, ^26^, ^27^, ^29^, ^30^, ^31^, ^32^ including African lions. To the authors' knowledge, however, newer commercially available immunochromatography assays, in particular those employing the FIV‐Fca transmembrane glycoprotein (gp40) as the capture antigen, have not yet been evaluated for their ability to detect antibody production due to FIV infection in non‐domestic felids.

The objective of this study was to evaluate the performance of four commercially available FIV antibody test kits for the detection of FIV‐Ple lentivirus infection in African lions held in zoological parks throughout Australia to determine whether these PoC assays, which are designed for use in domestic cats, can be extrapolated for lentivirus diagnosis in some non‐domestic felids.

## Materials and methods

### Study population

This project utilised archived serum samples and clinical records collected by the Sydney School of Veterinary Science, the University of Sydney, between 2005 and 2017 from African lions held in nine Australian zoos (Table S1), as part of a broader study.^36^ Samples were collected at the zoo of origin by the attending veterinarian for the purposes of a routine health check. Blood was centrifuged at the zoo, separated and sent by overnight courier at 4°C to the University of Sydney where initial testing was performed on fresh serum using SNAP® FIV/FeLV Combo (IDEXX Laboratories, Westbrook, ME, USA). Serum was then stored in cryovials at −80°C for additional testing later. Clinical histories were available for all lions, and none of the individuals included in the study had been vaccinated against FIV (Fel‐O‐Vax® FIV, Boehringer Ingelheim Animal Health Australia).

### Confirmation of SNAP® FIV results using WB

To evaluate the suitability of the SNAP® assay as a proxy reference test for FIV diagnosis, a subset of recruited samples from individual lions for which residual sample was available was subjected to WB. Each sample was from a single lion collected at a single timepoint and a mix of SNAP® FIV‐positive and SNAP® FIV‐negative samples was included. WB has traditionally been considered the preferred reference test for FIV diagnosis to determine FIV‐infected or FIV‐uninfected status^6^, ^27^, ^28^, ^37^, ^38^, ^39^; however, it requires sophisticated technology (such as cell culture for virus propagation) which is not readily available in most zoological facilities and is therefore not a practical tool for rapid diagnosis and decision‐making in captive management settings.

The subset of available serum samples was sent on dry ice to the University of Zurich for WB, as described elsewhere.^37^, ^38^, ^39^, ^40^ Briefly, the original FIV isolate from California, USA (FIV Z2^1^) was propagated in the FL‐4 T‐lymphoblastoid cell line in Gibco™ RPMI 1640 Medium (Thermo Fisher Scientific) with glutamine (GlutaMAX™ Supplement, Thermo Fisher Scientific) and 10% foetal calf serum. FIV Z2 was then concentrated by ultracentrifugation and denatured with SDS‐mercaptoethanol. After electrophoretic separation in a 12% PAGE gel, the protein antigens were transferred to nitrocellulose membranes at a concentration of approximately 1 μg of gradient‐purified FIV Z2 per strip. Lion serum samples were diluted 1:50 in INA‐4 buffer, comprising 20 g gelatin, 12.11 g Tris, 11.69 g NaCl and 1 g Tween 20 per 2 L ddH_2_O, with a pH 7.4 adjusted by concentrated HCl. The samples were incubated with the strips overnight on a rocker platform at room temperature. A peroxidase‐labelled goat anti‐cat immunoglobulin G conjugate diluted 1:1000 in INA‐4 buffer was then added and samples incubated for a further 2 h. Finally, strips were carefully washed before the substrate was added.^39^

The WB result was considered positive if two bands with a molecular weight of 15,000 (p15) and 24,000 (p24) Dalton, respectively, were recognizable on the blotting strip.^37^ If both bands were absent, the sample was considered WB‐negative.^37^ Samples that yielded only one band, either p15 or p24, were judged as inconclusive.^37^, ^38^, ^39^, ^40^ A positive control (serum from an experimentally infected FIV‐positive domestic cat confirmed with WB, ELISA and virus isolation)^41^ and a negative control (buffer only) were run with each batch of testing.

### Performance of four other FIV PoC tests in African lions

Four other commercially available PoC FIV antibody detection kits were evaluated in 2017 using thawed lion serum; VetScan® Rapid (Abaxis, Union City, CA, USA), SensPERT® (VetAll Laboratories, Gyeonggi‐do, Republic of Korea), Anigen Rapid® (BioNote, Gyeonggi‐do, Republic of Korea) and Witness® (Zoetis Animal Health, Lyon, France). VetScan® Rapid and SensPERT® kits detect antibodies to FIV capsid protein (p24), while Anigen Rapid® and Witness® kits detect antibodies to FIV glycoprotein (gp40) (Table 1). The original VetScan® Rapid FIV test has been unavailable in Australia since 2019. All testing was performed in accordance with the manufacturer's instructions. Serum from a SNAP® FIV‐positive domestic cat was used as a positive control, and any colour development in the test window within the specified time frame was interpreted as a positive result.

**Table 1:** Target antigen and methodology of five PoC FIV antibody test kits used for the detection of lion lentivirus infection in captive African lions.

FIV antibody detection kit	Methodology	Target antigen		
		p15	p24	gp40
SNAP®	Lateral flow ELISA	●	●	
VetScan® Rapid	Lateral flow immunochromatography		●	
SensPERT®	Lateral flow immunochromatography		●	
Anigen Rapid®	Lateral flow immunochromatography			●
Witness®	Lateral flow immunochromatography			●

### Statistical analysis

Cohen's kappa coefficient (*κ*) was calculated to evaluate the level of agreement between SNAP® and WB (https://www.statology.org/cohens-kappa-excel/), with 0 indicating no agreement and 1 indicating perfect agreement between the two assays.

The sensitivity (Se), specificity (Sp), positive predictive value (PPV) and negative predictive value (NPV) of each of the four PoC assays were calculated as follows: using the SNAP® FIV result as the true result: Se = ‘number of true positives’/(‘number of true positives’ + ‘number of false negatives’); Sp = ‘number of true negatives’/(‘number of false positives’ + ‘number of true negatives’); PPV = ‘number of true positives’/(‘number of true positives’ + ‘number of false positives’); NPV = ‘number of true negatives’/(‘number of true negatives’ + ‘number of false negatives’). Two‐sample binomial tests were used to compare the sensitivity and specificity of the four assays, and *p* < 0.05 was considered statistically significant.

Microsoft Excel® (Microsoft, Redmond, WA, USA) was used for data storage and calculations.

### Animal ethics

No ethical approval was required for this study as it utilised surplus archived samples which had been collected historically for routine diagnostic purposes. No additional samples were collected for the study.

## Results

### Study population

Forty‐eight individual African lions were included in the study. The cohort comprised 16 SNAP® FIV‐positive (10 male, 6 female) and 32 SNAP® FIV‐negative (14 male, 18 female) lions. Lions ranged from 4 months to 22 years of age at time of testing and the median age at the first test was 8.5 years. The median age of lions at their first FIV‐positive test was 11 years (range 3–22 years) and the median age of FIV‐negative lions was 8 years (range 4 months to 21 years).

### Confirmation of SNAP® FIV results using WB

Twenty‐six serum samples from 3 SNAP® FIV‐positive and 23 SNAP® FIV‐negative individuals were evaluated using WB. All SNAP® FIV‐positive samples (3/3) tested WB‐positive and 22/23 SNAP® FIV‐negative samples tested WB‐negative (Figure 1). One SNAP® FIV‐negative sample demonstrated reactivity with p24 but not p15 (defined as inconclusive) and was excluded from kappa analysis. Findings from the SNAP® and WB assays, therefore, demonstrated perfect agreement in lions with a SNAP® FIV‐positive result indicating FIV infection and a SNAP® FIV‐negative result indicating lack of FIV infection (*κ* = 1).

**Figure 1:**
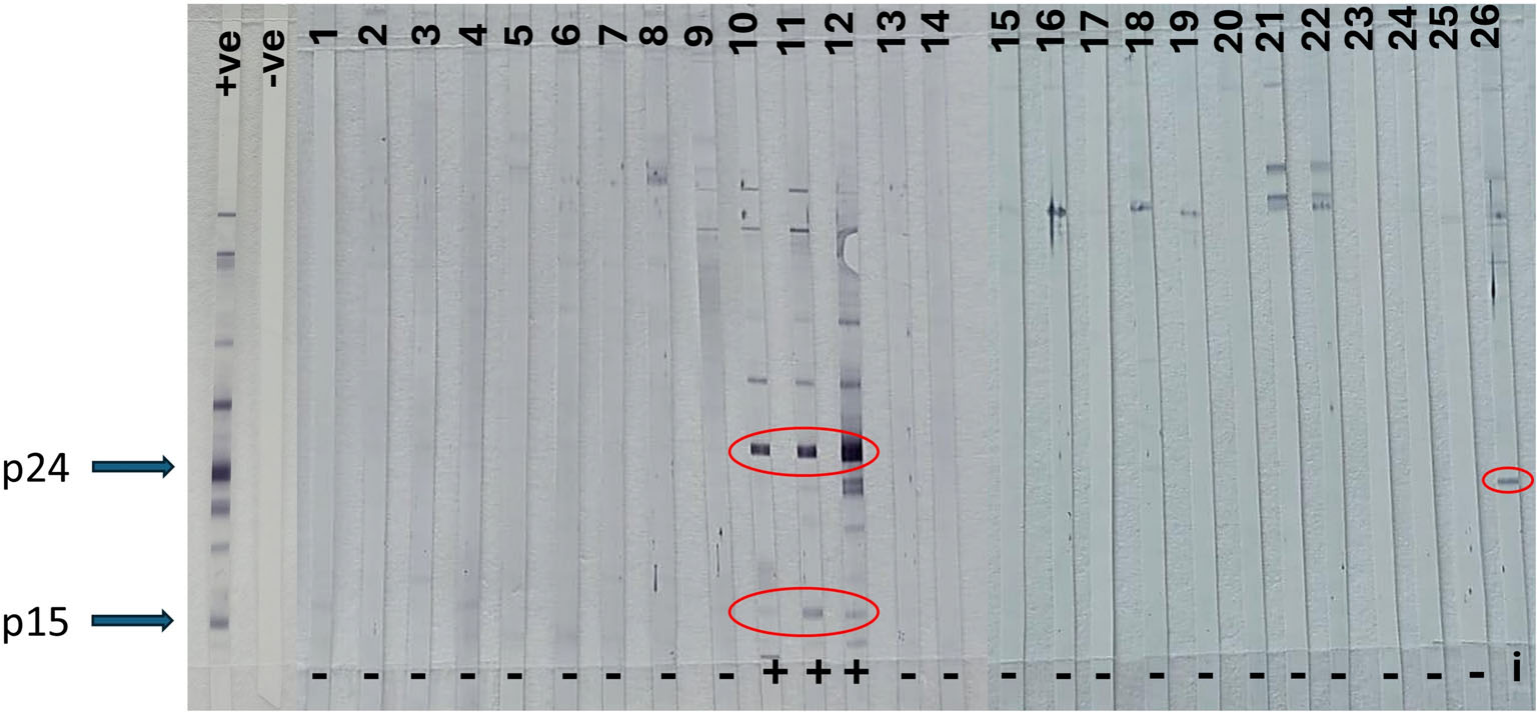
Western blot analysis of serum from 26 captive African lions held in zoological facilities in Australia. A FIV‐positive (+) result was indicated by reactivity against both matrix protein (p15) and capsid protein (p24). Reactivity against either p15 or p24 only was defined as an inconclusive (i) result. A lack of reactivity against both p15 and p24 was declared a FIV‐negative (−) result. FIV‐positive (+ve) and FIV‐negative (−ve) controls are included.

### Performance of the four other FIV PoC tests in African lions

Forty‐eight serum samples (including the 26 samples on which WB had been performed) were evaluated using the four PoC FIV antibody detection kits (Table 2). All FIV‐infected serum samples (16/16) tested positive using VetScan® Rapid, whereas only four samples returned positive results with SensPERT®. Zero samples tested positive using the Anigen Rapid® and Witness® assays. All FIV‐uninfected serum samples (32/32) tested negative using the SensPERT®, Anigen Rapid® and Witness® assays, whereas one FIV‐uninfected sample tested falsely positive with the VetScan® Rapid test. There were significant differences in the sensitivities of the four PoC FIV antibody detection assays, with VetScan® Rapid significantly more sensitive for diagnosing FIV infection than SensPERT®, Anigen Rapid® and Witness® (*p* < 0.001). All four assays demonstrated similar levels of specificity (Table 3).

**Table 2:** Results of four PoC FIV antibody test kits evaluated for the detection of lion lentivirus infection in African lions held in captivity in Australian zoos (*n* = 48 serum samples; comprising 16 SNAP® FIV‐positive and 32 SNAP® FIV‐negative samples).

	SNAP® (p15/p24)	VetScan® Rapid (p24)	SensPERT® (p24)	Anigen Rapid® (gp40)	Witness® (gp40)
True +ve	16	16	4	0	0
False + ve	0	1	0	0	0
True −ve	32	31	32	32	32
False −ve	0	0	12	16	16
Se	100	16/16 = 100% (79.4–100)	4/16 = 25.0% (7.3–52.4)	0/16 = 0% (0–20.6)	0/16 = 0% (0–20.6)
Sp	100	31/32 = 96.9% (84.2–99.9)	32/32 = 100% (89.1–100)	42/42 = 100% (89.1–100)	42/42 = 100% (89.1–100)
PPV	100	16/17 = 94.1% (69.9–99.1)	4/4 = 100% (39.8–100)	0/0 = 0%	0/0 = 0%
NPV	100	31/31 = 100% (89.1–100)	32/44 = 72.7% (66.8–78.0)	32/48 = 66.7% (53.3–80.0)	32/48 = 66.7% (53.3–80.0)

SNAP® was used as the reference test. Confidence intervals (95%) are given in brackets. NPV, negative predictive value; PPV, positive predictive value; Se, sensitivity; Sp, specificity.

**Table 3:** Comparisons of sensitivity and specificity among four PoC FIV antibody test kits evaluated for the detection of lion lentivirus infection in captive African lions.

FIV antibody detection kits	Sensitivity	Specificity
	(*p‐*value)	(*p*‐value)
VetScan® Rapid vs. SensPERT®	100% vs. 25%	96.9% vs. 100%
	**(<0.001)**	(1)
SensPERT® vs. Anigen Rapid®/Witness®^a^	25% vs. 0%	100% vs. 100%
	(0.10)	(1)
VetScan® Rapid vs. Anigen Rapid®/Witness®^a^	100% vs. 0%	96.9% vs. 100%
	**(<0.001)**	(0.43)

a Witness® and Anigen Rapid® produced the same test results. Significant comparisons are in bold text.

## Discussion

PoC tests are essential for the rapid and accurate diagnosis of FIV infection in captive lions, where a positive result has significant implications for the management and prevention of viral transmission among these threatened felids. This investigation evaluated the ability of four commercially available PoC FIV antibody test kits to detect FIV infection in captive African lions, when compared with results from SNAP®. All four PoC test kits had comparable specificity but differing sensitivity, with VetScan® Rapid demonstrating significantly higher sensitivity when compared to the other three PoC assays. Our findings highlight both potentials and pitfalls of extrapolating diagnostic assays designed for use in domestic cats to their wild counterparts. Importantly, SNAP® and VetScan® Rapid FIV PoC test kits both demonstrated excellent performance in our study population and should therefore be the preferred choice for rapid diagnosis of FIV infection in African lions held in Australian zoos.

We utilised SNAP® as the reference test as it has been a mainstay for PoC FIV diagnostics in domestic cats for decades, with sensitivity and specificity of up to 100% and 97%, respectively, in FIV‐unvaccinated cats,^22^ although some investigators have reported considerably lower values and discordance with WB due potentially to the presence of novel circulating FIV isolates.^39^ Furthermore, SNAP® is more easily adapted to field and zoo conditions than other more technically demanding assays such as WB.

We first evaluated SNAP® against a traditional reference test to verify it as an accurate diagnostic tool to detect FIV infection in African lions and a suitable confirmatory test against which the newer PoC assays could be evaluated. Virus isolation is considered the ultimate reference test for lion lentivirus diagnosis; however, the nature of our sample bank (frozen stored serum) precluded its use for this investigation. WB is widely recommended as a confirmatory test for any domestic feline returning a positive or inconclusive FIV result on another assay^42^ and was the modality of choice for evaluating the SNAP® test. We observed perfect agreement (*κ* = 1) between SNAP® and WB, confirming that SNAP® is an accurate assay for determining FIV status in captive African lions who have not been vaccinated against FIV and was a proxy confirmatory test against which the newer PoC assays could be evaluated.

Significant differences in sensitivity were observed among the four PoC FIV antibody detection assays evaluated to detect FIV infection in captive African lions, when compared to the SNAP® test (Table 3). VetScan® Rapid successfully returned a positive result for all FIV‐infected serum samples. In contrast, Anigen Rapid® and Witness® were unable to detect a single FIV‐positive sample, and SensPERT® was only able to detect 4/16 FIV‐positive samples. Although the role of FIV infection in causing clinical disease in African lions is controversial,^15^ misdiagnosing FIV‐infected lions with PoC testing (i.e., false‐negative results) could have important consequences for housing and management. The differences observed among the PoC tests may be related to the differing viral capture antigens employed (Table 1), since all four assays evaluated utilised the same methodology (colloidal gold lateral flow immunochromatography). The VetScan® Rapid and SensPERT® kits both feature the capsid protein (p24) as their target antigen, whereas transmembrane glycoprotein (gp40) is the target antigen for the Anigen Rapid® and Witness® assays. Our reference test, SNAP®, is a lateral flow ELISA which employs the matrix protein (p15) and capsid protein (p24) as its capture antigens (Table 1). p24 is encoded by the FIV *gag* gene,^43^ and is the first viral protein against which antibodies are developed in domestic cats experimentally infected with FIV.^44^ Furthermore, *gag* genes are highly conserved among lentiviruses, including HIV^45^ and FIV‐Fca,^44^ suggesting that p24 may also be conserved in FIV‐Ple isolates. In contrast, lentiviral *env* genes (of which gp40 is a product) are typically heterogeneous, with considerable variation among HIV isolates.^45^ Indeed, Calzolari and colleagues observed essentially no cross‐reactivity between FIV‐positive lion sera and recombinant FIV‐Fca transmembrane glycoprotein in an ELISA, and variable reactivity with naturally infected domestic cat sera, suggesting that reactivity may be specific for the infective or closely related isolates.^38^ Our findings confirm that the presence of FIV capsid protein p24 as target antigen is important for detecting lentiviral infection in African lions and that gp40 is not an informative antigen for this purpose.

Despite the employment of the same capture antigen (p24) and methodology (immunochromatography), there was a significant difference between the sensitivity of the VetScan® Rapid and SensPERT® assays (*p* < 0.001, Table 3), with the SensPERT® kit returning a higher number of false negative results (12/16) than the VetScan® Rapid kit (0/16). There are several potential reasons for this. First, it is possible that the thresholds at which the kits are set vary; that is, a greater level of anti‐p24 antibodies is required to result in a colour change to the test strip of the SensPERT® compared with the VetScan® Rapid assay. Indeed, many of the positive results obtained with the SensPERT® kits were ‘faint positives’, and it is possible that the false negative samples contained anti‐p24 antibodies at levels below the test threshold. Further investigation of this would involve quantitative anti‐p24 antibody testing of the FIV‐positive serum samples, for example using an anti‐p24 microsphere immunoassay.^46^, ^47^, ^48^ Second, the capture antigen may be produced or purified differently for each of the kits. The choice of expression system and purification route can markedly influence antigen structure and immunoreactivity.^49^, ^50^ Recombinant viral proteins expressed in prokaryotic systems often lack the post‐translational modifications seen in eukaryotic or mammalian‐cell–derived antigens, which can alter the presentation of key epitopes and reduce antibody‐binding affinity.^51^ Third, differences in the three‐dimensional conformation of the p24 antigen between kits may also contribute to variability in assay sensitivity. Antibody recognition of lentiviral capsid proteins is strongly dependent on correct conformational epitope presentation, as demonstrated by structural studies mapping monoclonal antibody‐binding sites on HIV‐1 p24.^52^ FIV p24 itself shows condition‐dependent conformational and assembly behaviour,^53^ supporting the plausibility that subtle alterations in folding or higher‐order structure of the capture antigen could reduce its reactivity with FIV‐positive lion sera. Thus, if the p24 antigen used in these kits is produced, processed or presented differently, this could potentially result in sufficient variation to affect reactivity between the capture antigen and FIV‐positive lion sera. Unfortunately, details regarding the exact recombinant antigens or peptide epitopes used in the commercial assays tested are proprietary and are not disclosed in manufacturers' product inserts, which we recognise as a possible limitation of this study.

We identified a single SNAP® FIV‐negative sample which reacted with WB to p24 only (i.e. not to p15). This inconclusive result may have indicated that the individual was in the very early phase of infection, as p24 is the first viral protein against which antibodies are developed in domestic cats experimentally infected with FIV.^44^ However, we were unable to perform the recommended serial testing 2–3 months later to assess for delayed reactivity to p15,^39^ due to the opportunistic nature of our serum database and lack of subsequent samples. Alternatively, this result could have indicated infection with a strain of FIV with sufficiently different p15 antigen to those used as capture antigens in WB and SNAP® that anti‐p15 antibodies could not be detected by either test. Frankenfeld and colleagues^39^ also identified a number of WB‐positive (p15 and p24) domestic cats whose infections were undetectable by SNAP® and hypothesised that it could be due to the presence of novel FIV isolates in their sample population, as many of the cats in question were imported or had a travel history. Viral genome sequencing of the lion FIV isolates would have been valuable for further consideration of this hypothesis; however, a lack of appropriate sample (anticoagulated whole blood or plasma) meant that this was beyond the scope of our investigation. Finally, the serum antibody concentrations may have been below the detection threshold for SNAP® and/or the antigen concentration higher on the WB than the PoC assay, which could explain the negative SNAP® result despite the WB‐confirmed presence of anti‐p24 antibodies in the sample. Again, anti‐p24 antibody quantification^46^, ^47^, ^48^ of the inconclusive serum sample would have been informative but was not within the remit of this study.

As with many studies on endangered species, the present investigation was unfortunately underpowered as a product of the high conservation value of lions, the impracticality of obtaining additional samples without sedation and restraint and the use of an archived serum biobank for retrospective analysis. Consequently, additional samples were unavailable to investigate findings further, including the single discordant SNAP®/WB result. Similarly, testing all samples by WB would have been preferable, but was precluded by the limited volume of archived serum available. Finally, because only serum samples were available, complementary investigations requiring cellular material, such as proviral PCR and viral genomic sequencing, could not be performed. These limitations are important considerations when interpreting our findings but were unavoidable given the study population and the retrospective nature of the investigation.

## Conclusions

PoC test kits form an integral component of FIV diagnosis in both domestic and non‐domestic felids. In the absence of FIV subtype‐specific assays, tests designed for use in domestic cats are commonly extrapolated for use in their wild counterparts; however, considerable genomic variation exists between domestic and non‐domestic FIV subtypes as a result of extensive host‐pathogen co‐evolution, and this was reflected in the variable performance of the commercially available assays. Our investigation indicates that FIV‐Fca p24 can be an informative antigen for the detection of FIV infection in captive African lions using some brands of FIV kits, consistent with its high immunogenicity and conserved nature among other species affected by this lentivirus. In addition, we identified that anti‐gp40 antibodies from FIV‐positive African lions are not detected by kits using FIV‐Fca gp40 as a capture antigen, suggesting that FIV‐Fca gp40 is not useful for FIV diagnosis in non‐domestic felids. Our findings have implications for captive endangered felids, in which FIV must be accurately diagnosed and individuals managed appropriately to minimise viral transmission.

## Conflicts of interest and sources of funding

The authors declare no conflicts of interest.

## Supporting information


**Table S1.** Origin and number of individual African lions (*Panthera leo*) included in this study. ACT = Australian Capital Territory; NSW = New South Wales; NT = Northern Territory; QLD = Queensland; VIC = Victoria; WA = Western Australia.

## Data Availability

The data that support the findings of this study are available from the corresponding author upon reasonable request.
