# Supplementary material for: Performance of point‐of‐care tests for diagnosing feline immunodeficiency virus infection in captive African lions ( Panthera leo )
Source: Aust Vet J. 2026 Sep 9;104(11):812–8. doi: 10.1111/avj.70137 (PMC13723756; doi:10.1111/avj.70137)
Supplement: Supplementary file 1 [file AVJ-104-812-s001.docx]

**Supplementary Data**

**Table S1.** Origin and number of individual African lions (*Panthera leo*) included in this study. ACT = Australian Capital Territory; NSW = New South Wales; NT = Northern Territory; QLD = Queensland; VIC = Victoria; WA = Western Australia.

| **Facility** | **State** | **No. lions tested** |
| --- | --- | --- |
| National Zoo and Aquarium | ACT | 2 |
| Mogo Zoo | NSW | 14 |
| Taronga Zoo | NSW | 4 |
| Western Plains Zoo | NSW | 9 |
| Crocodylus Park | NT | 1 |
| Darling Downs Zoo | QLD | 3 |
| Melbourne Zoo | VIC | 6 |
| Werribee Open Range Zoo | VIC | 4 |
| Perth Zoo | WA | 5 |
|  | TOTAL | 48 |
